# Targeting LncRNA HOTAIR suppresses cancer stemness and metastasis in oral carcinomas stem cells through modulation of EMT

**DOI:** 10.18632/oncotarget.21614

**Published:** 2017-10-07

**Authors:** Ming-Yi Lu, Yi-Wen Liao, Pei-Yin Chen, Pei-Ling Hsieh, Chih-Yuan Fang, Chia-Yu Wu, Ming-Liang Yen, Bou-Yue Peng, Dayen Peter Wang, Hsin-Chung Cheng, Ching-Zong Wu, Yung-Hsun Shih, Duen-Jeng Wang, Cheng-Chia Yu, Lo-Lin Tsai

**Affiliations:** ^1^ School of Dentistry, Chung Shan Medical University, Taichung, Taiwan; ^2^ Department of Dentistry, Chung Shan Medical University Hospital, Taichung, Taiwan; ^3^ Institute of Oral Sciences, Chung Shan Medical University, Taichung, Taiwan; ^4^ Department of Dentistry, Taipei Municipal Wanfang Hospital, Taipei, Taiwan; ^5^ School of Dentistry, College of Oral Medicine, Taipei Medical University, Taipei, Taiwan; ^6^ Department of Dentistry, Taipei Medical University Hospital, Taipei, Taiwan

**Keywords:** oral squamous cell carcinomas, long non-coding RNA, HOTAIR, cancer stem cells, mesenchymal

## Abstract

Increasing evidence indicates that long non-coding RNAs (lncRNAs) regulate diverse cellular processes, such as cell growth, apoptosis and tumorigenesis. However, the functional roles of lncRNAs and mechanistic analysis of their interplays with oncogenic pathways in oral cancer remain largely unknown. In the current study, we examined the significance of lncRNA HOTAIR (HOX transcript antisense RNA) in tumor progression of oral squamous cell carcinomas (OSCC). We found the expression of HOTAIR was upregulated in tumor tissues, especially in the metastatic samples. And it was also observed in metastatic OSCC cell lines. Silence of HOTAIR in oral carcinomas stem cells (OCSC) significantly inhibited their cancer stemness, invasiveness and tumourigenicity in xenotransplantation models. By contrast, overexpression of HOTAIR in OSCC enhanced their metastatic potential and epithelial-mesenchymal transition (EMT) characteristics. And we showed that the expression of HOTAIR was positively related to mesenchymal markers and inversely correlated with epithelial marker in clinical samples. Moreover, Kaplan-Meier survival analysis suggested that high level of HOTAIR was a strong predictor of poor survival in OSCC patients. Collectively, our data demonstrated that HOTAIR-mediated cancer stemness and metastasis are associated with the regulation of EMT and HOTAIR may serve as a therapeutic target in OSCC.

## INTRODUCTION

Oral squamous cell carcinoma (OSCC) is a type of head and neck cancers and represents around 90% of all malignant neoplasms of the oral cavity [1]. Despite the general decrease in the incidence of OSCC due to the reduced tobacco and arena nut usage, the survival rate has not drastically changed over the past few decades and the 3-year overall survival was less than 30% in advanced patients with level IV/V metastasis [2, 3]. In addition, cumulative evidence has indicated that failure of the conventional treatments might be attributed to cancer stem cells (CSCs), which possess tumor-initiating capacity and appear to play crucial roles in metastasis, relapse and chemo/radio-resistance. Indeed, CSCs have been shown to be implicated in the metastasis and chemoradioresistance in OSCC [4–6]. As such, eradicating CSCs by efficient targeting agents may be a novel therapeutic avenue for oral carcinogenesis.

Recent studies in cancer research have attempted to provide clarity to the mechanisms underlying the regulation of CSCs and uncover how these are linked to treatment effect. Among various factors that influence CSCs, non-coding RNAs have gained great attention. Non-coding RNA can be divided into small non-coding RNAs (such as microRNAs) and long non-coding RNAs (lncRNAs) based on the length of less or more than 200 nucleotides, respectively [7]. And numerous studies have shown that lncRNAs are involved in cancer progression [8–13] and drug resistance [14]. HOX antisense intergenic RNA (HOTAIR) is one of the well-known oncogenic lncRNAs. It has been demonstrated that HOTAIR was up-regulated in cancer tissues, especially in head and neck squamous cell carcinoma (HNSCC) [15–17]. In regard to oral cancer, a multiple studies have suggested that HOTAIR was highly expressed in OSCC tissues and facilitated the growth of OSCC cells via reduction of cell apoptosis [18, 19]. Moreover, it was found to regulate tumor invasion and metastasis [15, 20, 21] as well as contribute to chemoradioresistance [22, 23] in various cancers. The expression level of HOTAIR was correlated with tumor size and clinical stage in OSCC [19, 24] and HOTAIR has been suggested as a prognosis factor for HNSCC [16, 17, 25]. Recently, studies have demonstrated that HOTAIR expression was associated with the characteristics of CSCs [26, 27]. Nevertheless, HOTAIR-mediated regulation of oral carcinomas stem cells (OCSC) still remains to be elucidated.

It is well known that cancer cells exploit epithelial-to-mesenchymal transition (EMT), the capacity of epithelial cells to acquire mesenchymal traits, to invade and metastasize. EMT has been found to be a crucial step for generation of CSCs [28] and related to cancer aggressiveness [29]. Previously, it was reported that HOTAIR was required for EMT and stemness maintenance in colon and breast cancer cell lines [30]. In the current study, we aimed to investigate the role of HOTAIR in the oncogenicity of OCSC and whether it regulates these characteristics via EMT process. We examined the cancer stemness and invasiveness *in vitro* and tumorigenicity in xenotransplantation model and demonstrated that modulation of HOTAIR could be considered for the development of CSC-targeted therapy in oral carcinogenesis.

## RESULTS

### The up-regulation of HOTAIR expression in lymph node metastatic OSCC cell lines and OSCC patients

**Figure 1:**
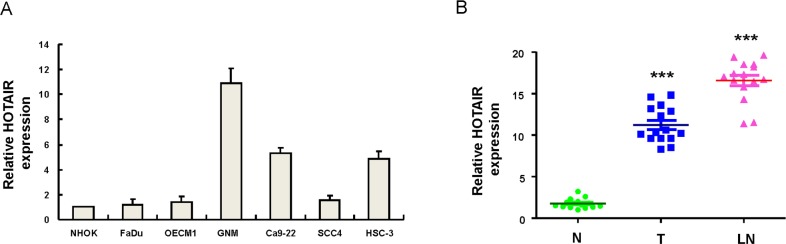
Relative expression of HOTAIR in 6 OSCC cell lines and OSCC patients with localized and metastatic tumors **(A)** The expression level of HOTAIR was markedly elevated in lymph node metastatic GNM cell line compared to normal human oral keratinocytes (NHOK); **(B)** Adjacent noncancerous matched tissues (NCMT; n=15), and paired tissue samples from tumor (T; n=15) as well as lymph node metastatic (LN; n=15) lesions in OSCC patients were subjected to analysis for the expression levels of HOTAIR. The expression level of HOTAIR in the samples of lymph node metastatic tumors (LN) was further upregulated compared with localized tumors (T). ^***^p <.05 compared to non-tumor (N) tissue.

To understand the expression of HOTAIR in OSCC cell lines, the endogenous transcript level of HOTAIR in six established OSCC cell lines and normal human oral keratinocytes (NHOK) was examined by real-time RT-PCR analysis. As shown in Figure 1A, the expression level of HOTAIR was highly detectable in lymph node metastatic OSCC cell line (GNM) compared to NHOK cells. To validate the aberrant expression of HOTAIR in clinical specimens, we collected samples of non-tumor (N), local tumor (T) and lymph node (LN) tissues from OSCC patients and these samples were subjected to real-time RT-PCR analysis. The expression of HOTAIR was elevated in the tumor samples (Figure 1B) compared with non-tumor tissues from the same patients. Also, a further up-regulation of HOTAIR was observed in metastatic lymph nodes when compared with localized tumors (Figure 1B).

### Down-regulation of HOTAIR expression reduces stemness features in oral cancer stem cells

**Figure 2:**
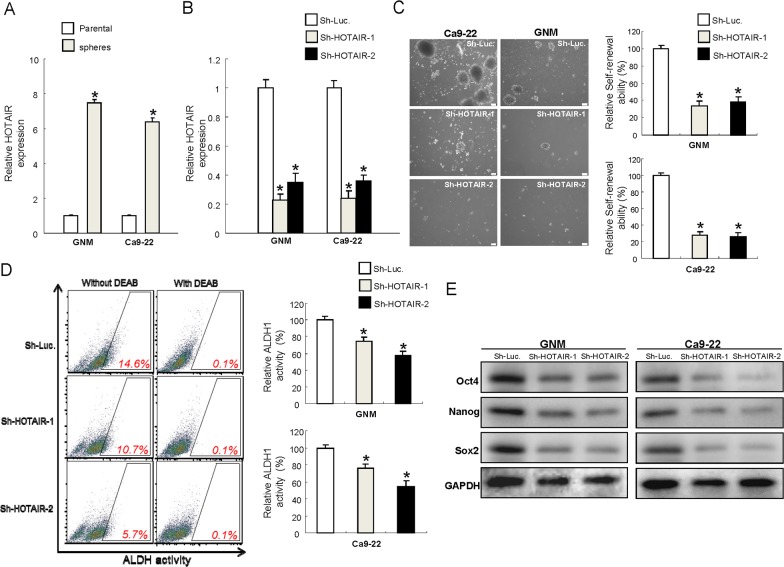
Reduced expression of HOTAIR in OSCC-CSCs suppresses the signatures of stemness **(A)** The expression of HOTAIR was significantly elevated in the OSCC-CSCs, ^*^p <.05 compared to parental cells; **(B)** The silencing effect of HOTAIR by lentiviral-mediated knockdown was validated by RT-PCR; Down-regulated expression of HOTAIR inhibited the self-renewal ability **(C)**, ALDH1 enzymatic activity **(D)** and expression level of stemness markers in OSCC-CSCs, **(E)** Data shown as mean ± SD of three independent experiments. ^*^p <.05 compared to control lentiviral vector (sh-Luc).

Previously, oralspheres from nonadhesive culture system have been shown to possess CSCs properties [31]. And ALDH1 has been shown to be sufficient to serve as a single and specific marker for identification of head and neck squamous cell carcinoma cancer stem cells [32, 33]. We selected two cell lines, GNM and Ca9-22, which exhibited significantly increased level of HOTAIR (Figure 1) to conduct the following experiments. Results from quantitative RT-PCR analysis confirmed that HOTAIR levels were even higher in oralspheres compared with parental cells (Figure 2A). To further investigate whether HOTAIR plays a role in maintaining CSCs hallmarks, loss-of-function mutation was generated in GNM and Ca9-22 sphere-forming oral cancer stem cells using small hairpin RNA targeting HOTAIR (sh-HOTAIR-1 and sh-HOTAIR-2), and lentiviral vector expressing shRNA against luciferase (sh-Luc) was used as control. Real-time RT-PCR showed that the expression level of HOTAIR was markedly reduced in sh-HOTAIR cells (Figure 2B). Knockdown of HOTAIR in OSCC-CSCs suppressed their secondary sphere-forming ability (Figure 2C) and reduced ALDH1 activity (Figure 2D). Moreover, the expression of various pluripotent stemness markers was also decreased (Figure 2E and Supplementary Figure 1).

### Reduced HOTAIR expression represses the metastatic potential of OSCC-CSCs *in vitro* and oncogneicity *in vivo*

**Figure 3:**
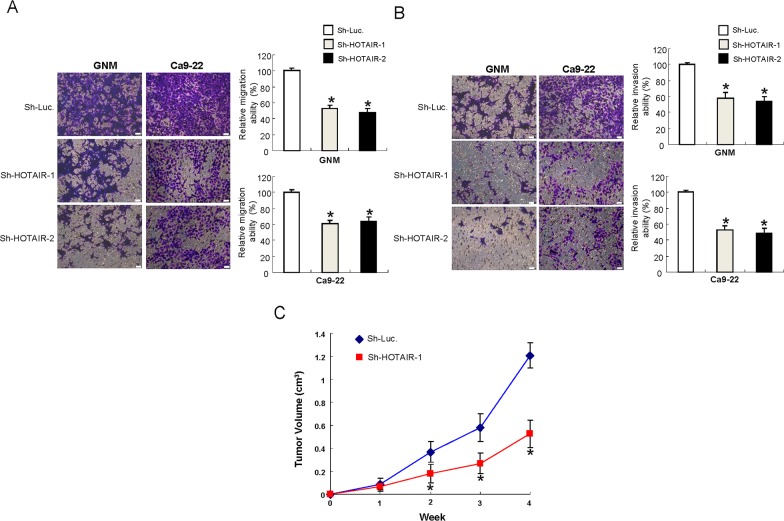
Inhibition of the metastatic potential *in vitro* and oncogneicity *in vivo* after silencing HOTAIR expression in OCSCs **(A)** Migration and **(B)** invasion abilities of OCSCs were attenuated after downregulation of HOTAIR, data shown as mean ± SD of three independent experiments. ^*^p <.05 compared to control lentiviral vector (sh-Luc). **(C)** Nude mice were subcutaneously injected with sh-HOTAIR-1 or sh-Luc transfected OSCC-CSCs, and the mice were monitored for 4 weeks. OSCC-OCSCs with reduced HOTAIR expression showed smaller tumor size, indicating the slower tumor growth in xenotransplantation model.

Metastasis remains a significant challenge for cancer treatment. As such, we sought to examine the importance of HOTAIR in metastatic activities and assess its contribution in tumorigenesis *in vivo*. Our data suggested that down-regulation of HOTAIR inhibited the migration (Figure 3A) and invasion (Figure 3B) capacities of both OSCC-CSCs. Furthermore, mice transplanted with HOTAIR-knockdown tumor cells exhibited significantly slower tumor growth (Figure 3C).

### Metastasis properties in OSCC are enhanced following overexpression of HOTAIR

**Figure 4:**
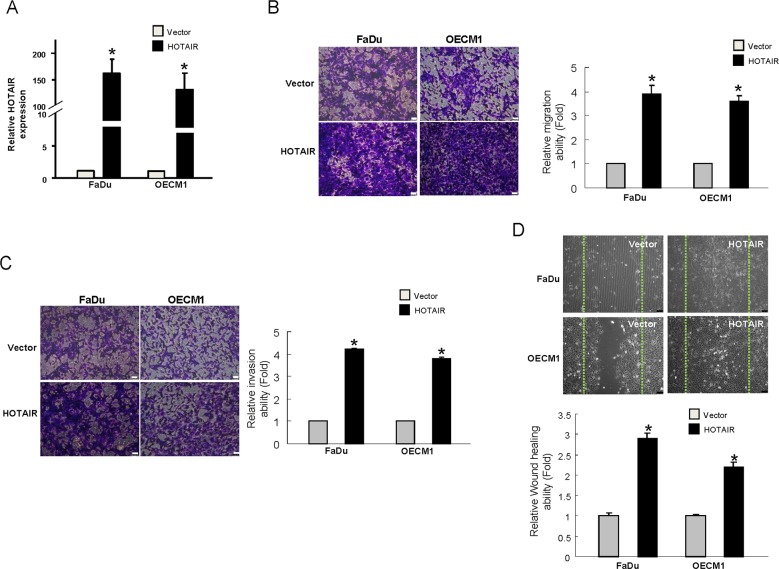
Elevation of the metastatic properties in OSCCs with overexpressed HOTAIR **(A)** The expression of HOTAIR has been confirmed by RT-PCR and shown to be significantly reduced in the OSCCs; **(B)** Migration and **(C)** invasion as well as **(D)** wound healing abilities were increased. Data shown as mean ± SD of three independent experiments. ^*^p <.05 compared to control lentiviral vector (sh-Luc)

Since HOTAIR seemed to be implicated in tumor progression, we chose the OSCC with low level of HOTAIR, FaDu and OECM-1, to generate stable HOTAIR-overexpressing cells through lentiviral-mediated transduction in order to verify this hypothesis. As shown in Figure 4A, both HOTAIR-overexpressing OSCC cell lines displayed elevated level of HOTAIR. Next, we evaluated the invasiveness of these HOTAIR-overexpressing OSCC cells. As expected, the upregulated HOTAIR increased their capacities to migrate (Figure 4B) and invade (Figure 4C) as well as enhanced the wound healing ability (Figure 4D).

### Diminished EMT traits in OSCC-CSCs by targeting HOTAIR

**Figure 5:**
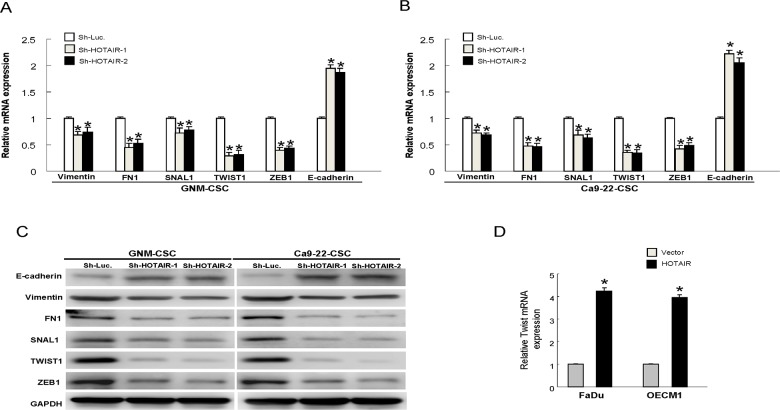
Expression of HOTAIR promotes the EMT characteristics The gene expression of the mesenchymal markers, including vimentin, FN1, Snail, Twist and ZEB1, were reduced and epithelial marker E-cadherin was increased following down-regulation of HOTAIR in GNM-CSCs **(A)** and Ca9-22-CSCs **(B)**; The similar changes of protein expression were observed in OSCC-CSCs **(C)**; **(D)** The expression of Twist was enhanced in HOTAIR-overexpression OSCCs. Data shown as mean ± SD of three independent experiments.

It is well known that CSCs exerted high tumorigenic, chemo-radioresistant, metastatic properties coupled with gain of epithelial-mesenchymal transition (EMT) characteristics. And it has been found that HOTAIR involved in the down-regulation of E-cadherin [24]. Therefore, we investigated whether oncogenic effect of HOTAIR was associated with the regulation of EMT in OSCC-CSCs. Indeed, results from real-time PCR and western blot analyses demonstrated the decreased expression of mesenchymal-like markers (vimentin, FN1, Snail, Twist and ZEB1) and increased expression of epithelial protein (E-cadherin) following knockdown of HOTAIR in OSCC-CSCs (Figure 5A, 5B, 5C and Supplementary Figure 1). Additionally, overexpression of HOTAIR enhanced the level of mesenchymal-like marker (Twist) in both OSCC cells (Figure 5D).

### Clinical significance of HOTAIR in OSCC patients

**Figure 6:**
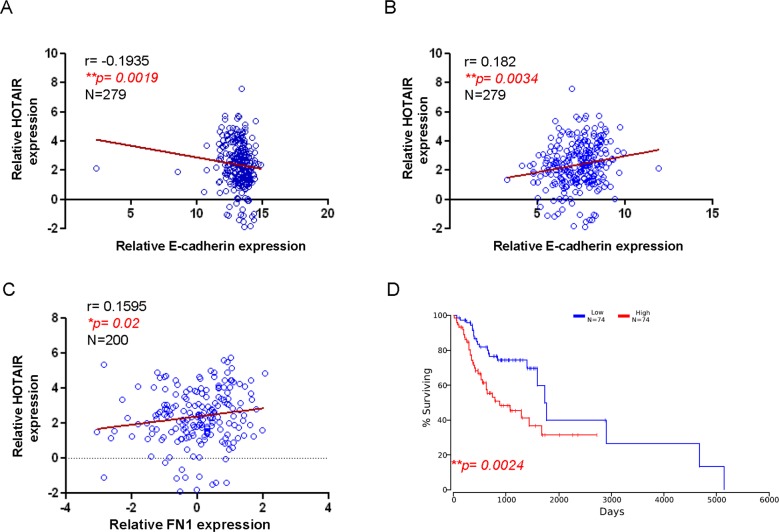
Correlation of HOTAIR with EMT markers and survival rate of OSCC patients HOTAIR negatively correlated with epithelial marker E-cadherin **(A)** but positively associated with mesenchymal markers, including Twist **(B)** and FN1 **(C)**. Kaplan-Meier survival analysis of OSCC patients from The Cancer Genome Atlas (TCGA) database demonstrated that higher HOTAIR expression led to poor survival **(D)** using Pearson's correlation coefficient.

In accordance with our findings, the expression of HOTAIR in clinical samples was negatively correlated to epithelial marker (E-cadherin) (Figure 6A) and positively associated with the mesenchymal-like markers (Twist and FN1) (Figures 6B and 6C). These results indicated that HOTAIR may contribute to CSCs characteristics via promoting EMT. Most importantly, Kaplan-Meier survival analysis of OSCC patients with high levels of HOTAIRshowed a reduced survival rate compared to low-expression subjects (Figure 6D) based on the Cancer Genome Atlas (TCGA) database.

## DISCUSSION

The clinical significance of HOTAIR has been summarized and discussed in multiple meta-analysis studies, suggesting that the expression of HOATIR is positively associated with an advanced clinical tumor stage, metastasis and worse prognosis [34, 35]. It has been shown that HOTAIR contributes to chemoresistance through PI3K/AKT/MRP1 [36], wnt/β-catenin [37] pathways or downregualtion of p21 [22] in other carcinomas. And it promotes cell proliferation by inhibiting miRNA-126 [36] or miRNA-218 [38] expression. Also, it controls cell cycle through functioning as a competing endogenous RNA to directly bind to miR-1 [39]. Interestingly, a recent study has demonstrated that exosomal HOTAIR induces human colon cancer cell line to form more colonospheres [40]. And it has been demonstrated that several cancer stem cell (CSC)-like traits were upregulated via induction of EMT after HOTAIR was enhanced in liver normal stem cells [41]. Theres results were in associated with previous studies showing that HOTAIR expression was related to various hallmarks of CSCs [20, 21]. Several studies have demonstrated that HOTAIR promotes CSCs growth through downregulation of SETD2 [26], regulation of EMT [27, 42] and stem cell markers [43].

It is well known that chromatin modifications are required for EMT and polycomb-repressive complex 2 (PRC2) is a key player in chromatin-repressive modification. In addition, HOTAIR has been identified as an assembling scaffold for enhancer of zeste homolog 2 (EZH2), an enzymatic subunit of PRC2 [44, 45]. EZH2 is frequently overexpressed in various cancers and is critical for cancer cell proliferation. It has been shown that various lncRNAs, such as LINC00511 or XIST, served as a modular scaffold of EZH2/PRC2 complexes and interacted and coordinated their localization, thereby contributing to cancer progression [46]. As for HOTAIR, it has been found that the expression of EZH2 was significantly decreased and the level of E-cadherin was enhanced in the HOTAIR knockdown OSCC cells [24]. They demonstrated that silence of HOTAIR reduced the binding of EZH2 on the promoter of E-cadherin [24]. Recently, HOTAIR has been shown to directly modulate the physical interaction between Snail and EZH2, thereby regulating the Snail-mediated EMT [47]. And EZH2 was also been proved to be required for Twist-induced EMT [48]. In associated with the finding that HOTAIR is epistatic to the master repressor of E-cadherin for EMT induction, we further observed a decrease in the expression of EMT markers in OSCC-CSCs following HOTAIR downregulation in the current study.

Previously, it has been shown that increased HOTAIR expression promoted tumor sphere formation in non-small cell lung cancer cells via upregulation of the stem cell-associated markers, including Sox2, Nanog, Oct3/4, c-Myc, β-catenin and Klf4 [49]. In breast cancer stem cells, HOTAIR has been shown to contribute to the proliferation, migration, colony formation, and self-renewal capacities through transcriptionally inhibiting miR-34a of breast CSCs, leading to upregulation of Sox2 [43]. Our previous work has demonstrated that Sox2-mediated CSCs property was associated with the regulation of EMT in OSCC-CSCs [6]. Investigation of the relationship between HOTAIR and stem cell marker, such as Sox2 in OSCC may provide another evidence to understand the HOTAIR-regulated features of OSCC-CSCs in the future.

In the present study, we found the upregulated expression of HOTAIR in OCSC, which was tightly associated with the metastatic features of OSCC. We demonstrated that overexpression of HOTAIR in OCSC enhanced cancer stemness and metastasis, whereas down-regulation of HOTAIR expression markedly attenuated the oncogenicity and invasiveness as well as tumor growth in xenograft nude mice. Additionally, decreased expression of HOTAIR in OCSC resulted in repressed EMT characteristics. These results indicated that HOTAIR-mediated regulation of OCSC was via modulating EMT markers and these findings showed that targeting HOTAIR may be a promising avenue for OSCC treatment.

## MATERIALS AND METHODS

### OSCC tissues acquirement and cell culture

Tissues were isolated from OSCC patients recruited in the Oral Medicine Center (Chung Shan Medical University Hospital, Taichung, Taiwan) with informed consent and the protocol was approved by Institutional Review Board of Chung Shan Medical University Hospital. Human primary 15 pairs of OSCC carcinoma (T) tissue, normal paired noncancerous matched tissues (N), as well as available lymph node (LN) metastatic lesions were obtained from surgical procedures sent to the pathology lab for frozen section diagnosis. Tumor tissues were microscopically screened to have >70% of their areas occupied by tumor cells; The remaining specimen (tumor, normal counterpart, and lymph node metastatic lesions) were snap frozen in liquid nitrogen and stored at −80°C for HOTAIR analysis. Six OSCC cell lines, including Fadu (hypopharyngeal squamous cell carcinoma carcinoma) [50], OECM-1 (gingival squamous cell carcinoma cells) [51], GNM (neck metastasis of gingival carcinoma) [52], Ca9-22 (gingival carcinoma) [53], SCC4 (tongue squamous cell carcinoma), HSC3 (tongue squamous cell carcinoma) cells [53] and primary normal human oral keratinocytes (NHOK) cells have been reported and were used in this study [5]. Briefly, Fadu and OECM-1 cells were in RPMI supplemented with 10% fetal bovine serum (FBS; Life Technologies, Carlsbad, CA, USA). Other cells were grown in Dulbecco's modified Eagle's medium (DMEM; Life Technologies, Carlsbad, CA, USA) supplemented with 10% FBS (Life Technologies). Cells were maintained in the appropriate growth medium at 37°C in a humidified atmosphere of 5% CO_2_.

### Quantitative real-time reverse-transcriptase (RT)-PCR

RT–PCR was performed as previously described [5] using SYBR green assay with specific primer sets (Applied Biosystems, Carlsbad, CA. USA). Total RNA was extracted from cells or tissues using Trizol reagent (Invitrogen). Superscript III first-strand synthesis system (Invitrogen) was used for cDNA synthesis according to the manufacturer's instruction. The reaction mix was incubated at 25°C for 10 min, 50°C for 50 min and 85°C for 5 min. GAPDH housekeeping gene was used as reference. Amplification and detection were carried out on an ABI StepOne™ Real-Time PCR Systems (Applied Biosystems, Carlsbad, CA, USA) for analysis of HOAIR and EMT-related markers (Supplementary Table 1).

### Lentiviral-mediated RNAi for silencing HOTAIR

The pLV-RNAi vector was purchased from Biosettia Inc. (Biosettia, San Diego, CA, USA). The method of cloning the double-stranded shRNA sequence is described in the manufacturer's protocol. Lentiviral vectors expressing shRNA that targets human HOTAIR (oligonucleotide sequence: Sh-HOTAIR-1:5’- AAAAGGAGTACAGAGAGAATAATTTGGATCCAAATTATTCTCTCTGTACTCC -3’;Sh-HOTAIR-2:5’AAAAGCTTCCTTGCTCTTCTTATTTGGATCCAAATAAGAAGAGCAAGGAAGC-3’) were synthesized and cloned into pLVRNAi to generate a lentiviral expression vector. Sh-Luc:5’-CCGGACTTACGCTGAGTACTTCGAA CTCGAGTTCGAAGTACTCAGCGTAAGTTTTTTG-3’ was utilized for experimental control. Lentivirus production was performed by co-transfection of plasmid DNA mixture with lentivector plus helper plasmids (VSVG and Gag-Pol) into 293T cells (American Type Culture Collection, Manassas, VA) using Lipofectamine 2000 (LF2000, Invitrogen, Calsbad, CA, USA) as described previously [54]. The lentivirus M.O.I titer is determined by flow cytometry (average of 5 × 10^4^ and 2 × 10^5^ TU/ml). To generate the stable cell lines, sub-confluent OSCC cells were infected with lentivirus in the presence of 8 μg/ml polybrene (Sigma-Aldrich, St Louis, MO, USA). The green fluorescence protein (GFP), which was co-expressed in lentiviral-infected cells, was served as a selection marker to indicate the successfully infected OSCCs. Stable pLV-RNAi expressed HNSCC cell lines were further purified by cell sorting with GFP positive cells.

### Overexpression of HOTAIR in OSCC cells

Human HOTAIR was cloned into pCDH1-MCS1-EF1-copGFP vector (System Biosciences, Cat. No: CD511A-1; Mountain View, CA, USA). Lentivirus production was performed as described above. The green fluorescence protein (GFP), which was co-expressed in lentiviral-infected cells, was served as a selection marker to indicate the successfully infected OSCCs. Stable HOTAIR-overexpressing OSCC cell lines were further purified by cell sorting with GFP positive cells. The pCDH1-MCS1-EF1-copGFP empty vector alone is utilized for experimental control.

### Secondary sphere formation assay

Cells were cultured in the modified DMEM/F-12 supplemented with N2 (R&D Minneapolis, MN, USA), 10 ng/mL epidermal growth factor (Invitrogen, Carlsbad, CA, USA), 10 ng/mL basic fibroblast growth factor (Invitrogen, Carlsbad, CA, USA), and penicillin/streptomycin at 10^3^ cells/ low-attachment six-well plate (Corning Inc., Corning, NY, USA). Cell density/ 10,000 cells were presented as the percentage of control [55].

### ALDH1 enzymatic activity assay

ALDEFLUOR assay kit (StemCell Technologies) was used to examine the ALDH1 activity according to manufacturer's instructions. N,N-diethylaminobenzaldehyde (DEAB) was used as a substrate and inhibitor for ALDH isoenzymes [56]. ALDH^+^ cells were analyzed by flow cytometry (FACSCalibur; BD Biosciences) to compare the fluorescence intensity with the DEAB-treated negative control using CellQuest software [6].

### Immunoblotting analysis

Cells were lysed in NP-40 buffer and protein concentration was determined using BCA protein assay kit (Thermo Fisher Scientific, Rockford, IL, USA). Samples were separated by 10% SDS-PAGE and wet-transferred to a PVDF membrane (Millipore, Billerca, MA, USA) followed by incubation with primary antibodies against E-cadherin (Santa Cruz. Biotechnology Inc., Santa Cruz, CA, USA; 1:500), vimentin (Santa Cruz; 1: 500), FN-1 (Santa Cruz; 1: 500), SNAIL1 (Cell Signaling Technology Inc., Danver, MA, USA; 1: 500), TWIST (GeneTex, Irvine, CA, USA; 1: 500), ZEB1(Santa Cruz; 1: 500) or GAPDH (GeneTex, Irvine, CA, USA; 1: 5000). After incubation with corresponding secondary antibodies, the immunoreactive bands were developed using an ECL-plus chemiluminescence substrate (Perkin-Elmer, Waltham, MA, USA) and captured by LAS-1000 plus Luminescent Image Analyzer (GE Healthcare, Piscataway, NJ, USA) [55].

### Migration and invasion assays

Cell migration and invasion assays were carried out as previously described [55]. The cells were seeded to the upper compartment at the density of 1 × 10^5^ in 250 μL serum-free medium and medium supplemented with 10% FBS was used as a chemoattractant in the lower chamber. After 24 h of incubation, the filter membrane was stained with 0.1% Crystal Violet. The cells were then visualized and counted from five different fields of 100-fold magnification under an inverted microscope.

### Imaging of tumor growth *in vivo*

All procedures involving animals were conducted in accordance with the institutional animal welfare guidelines of the Chung Shan Medical University. 5–6 weeks old immunodeficient nude mice (BALB/c-nu/nu mice) were used for the xenograft models. Sh-Luc-expressing and Sh-HOTAIR-1 knockdown GNM-OCSC (1 × 10^4^ cells/0.2 mL/mouse) and were injected subcutaneously into 6 mices. Tumor size measurement was performed using an IVIS50 animal imaging system (Xenogen Corp.). The volume was calculated (according to the following formula: [length × width2]/2), and then analyzed by Image-Pro Plus software. The animals were euthanized after 4 weeks [57].

### Wound healing assay

Cells were seeded into 6-well culture dishes. Wounds were introduced to the confluent monolayer of cells to create a denuded area using a sterile 200μL plastic pipette tip. Cell movement toward the center of the wound area was photographed under a microscope [58].

### HOTAIR and EMT markers expression analyses in OSCC patients

To examine the correlation between HOTAIR and EMT markers expression in OSCC cancer patients, the gene expression data were downloaded from websites of The Cancer Genome Atlas (TCGA) data portal (https://cancergenome.nih.gov/), and analyzed data using GraphPad Prism software. We applied Spearman correlation analysis to explore the relationship between HOTAIR and EMT markers expression. Kaplan-Meier analysis was performed to evaluate the survival distribution for the top 15% patients (with high expression) and the bottom 15% of patients (with low expression) from 496 TCGA OSCC tumor samples. The statistical significance of survival difference between groups was determined using the log-rank test.

### Statistical analysis

Statistical Package of Social Sciences software (SPSS; version 13.0) was used for statistical analysis. Student's *t* test or ANOVA analysis were used to determine statistical significance of the differences between experimental groups; *p* values less than 0.05 were considered statistically significant

## SUPPLEMENTARY MATERIALS FIGURE AND TABLE


