# Supplementary material for: Targeting LncRNA HOTAIR suppresses cancer stemness and metastasis in oral carcinomas stem cells through modulation of EMT
Source: Oncotarget. 2017 Oct 7;8(58):98542–52. doi: 10.18632/oncotarget.21614 (PMC5716749; doi:10.18632/oncotarget.21614)
Supplement: Supplementary file 1 [file oncotarget-08-98542-s001.pdf]

Targeting LncRNA HOTAIR suppresses cancer stemness and metastasis in oral carcinomas stem cells through modulation of EMT

SUPPLEMENTARY MATERIALS

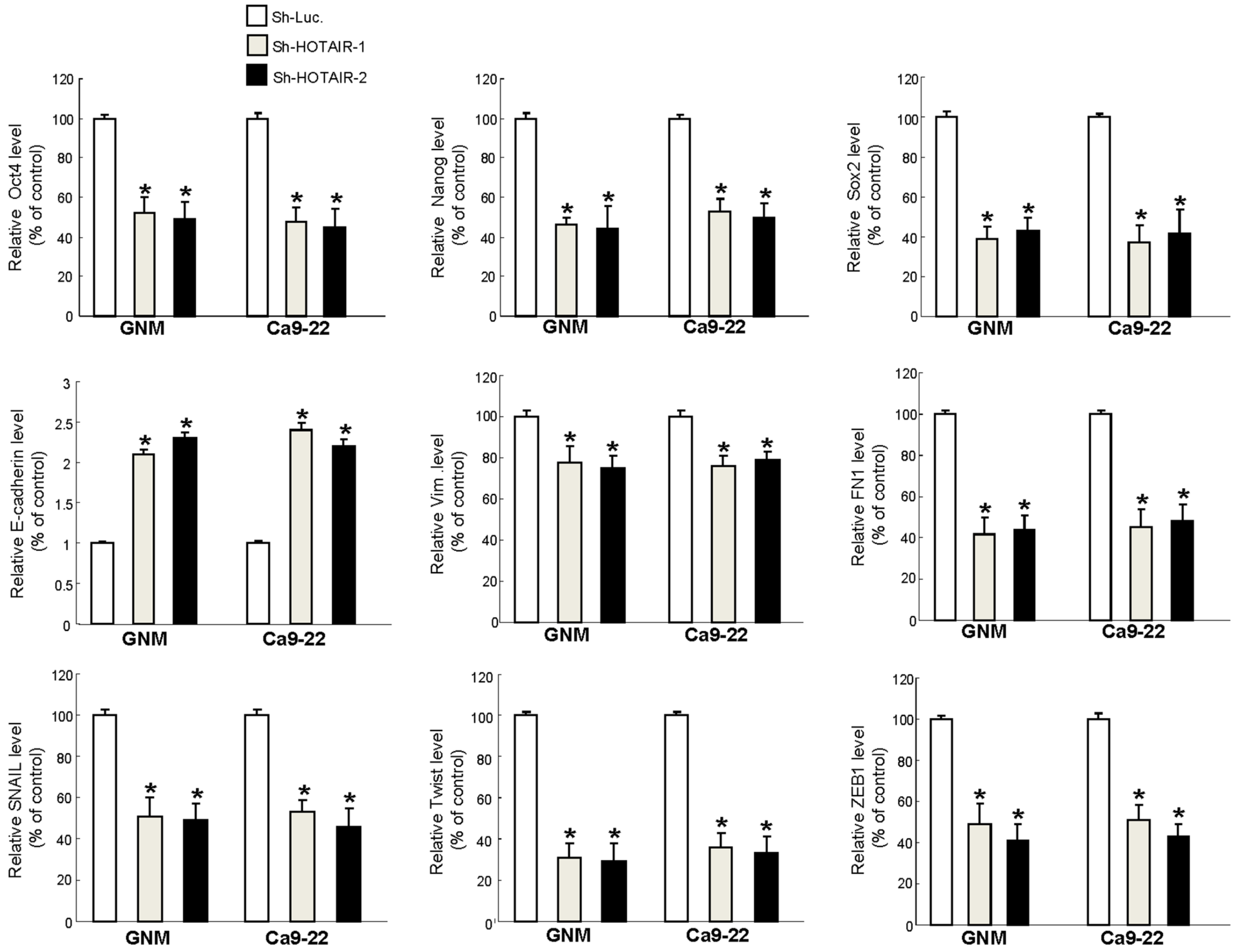

Supplementary Figure 1: Levels of indicated protein by western blotting experiments were measured by densitometer. The relative level of indicated protein expression was normalized against GAPDH and the control was set as 100%. Optical density values represent the mean ± SD. *Represents significant difference from control values with *p* <.05.

Supplementary Table 1: The sequences of the primers for quantitative RT-PCR

Gene	Primer Sequence (5' to 3')
HOTAIR	F: GGTAGAAAAAGCAACCACGAAGC
	R: ACATAAACCTCTGTCTGTGAGTGCC
E-cadherin	F: ATTCTGATTCTGCTGCTCTTG
	R: AGTCCTGGTCCTCTTCTCC
Vimentin	F: CAATGTTAAGATGGCCCTTG
	R: GGGTATCAACCAGAGGGAGT
Fibronectin	F: CCCAGACTTATGGTGGCAATTC
	R: AATTTCCGCCTCGAGTCTGA
Snail	F: GCAGCTATTTCAGCCTCCTG
	R: GTTCTGGGAGACACATCGGT
Twist	F: GGGAGTCCGCAGTCTTACGA
	R: AGACCGAGAAGGCGTAGCTG
ZEB1	F: AGCAGTGAAAGAGAAGGGAATGC
	R: GGTCCTCTTCAGGTGCCTCAG
GAPDH	F: CTCATGACCACAGTCCATGC
	R: TTCAGCTCTGGGATGACCTT
